# Quack‐induced dermatitis: A case report of two dermatitis artefacta induced by quacks advise

**DOI:** 10.1002/ccr3.8658

**Published:** 2024-03-10

**Authors:** Prajwal Pudasaini, Sadiksha Adhikari, Kinnor Das, Suraljit Gorai, Ruri D. Pamela

**Affiliations:** ^1^ Civil Service Hospital Kathmandu Nepal; ^2^ Gandaki Medical College Pokhara Nepal; ^3^ Apollo Clinic Silchar Assam India; ^4^ Apollo Multispecialty Hospitals Kolkata West Bengal India; ^5^ Department of Dermatology, Venereology & Aesthetic, Dr. Suyoto Hospital Ministry of Defense Jakarta Indonesia

**Keywords:** dermatitis Artefacta, psychodermatology, quack‐induced dermatitis, self‐inflicted lesions

## Abstract

This report delineates two instances of dermatitis artefacta (DA), a psychodermatological condition marked by self‐induced or exacerbated skin lesions. These cases, triggered by treatments from non‐qualified practitioners, highlight the critical need for healthcare professionals to discern the potential repercussions of unsound medical guidance.

## Graphical abstract

Quack‐induced dermatitis artefacta

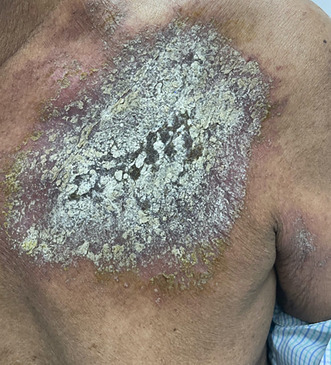

## 1 INTRODUCTION

Dermatitis artefacta (DA) is a psychodermatological entity with varied clinical phenotypes. Most often, patient denies their activities in the causation of this rare disease. Dermatitis artefacta has been linked to preceding medical and more often, psychological illnesses. Anxiety, phobia, stress, and underlying personality disorder have been implicated in the causation of this multimodal disease. Diagnosis can be made with good clinical acumen. (1) Many a times, DA can be caused by unscientific medications by quacks as well. Here, we report DA cases with varied clinical presentations and unique negligence.

## 2 CASE PRESENTATION

### 2.1 Case 1

**FIGURE 1:**
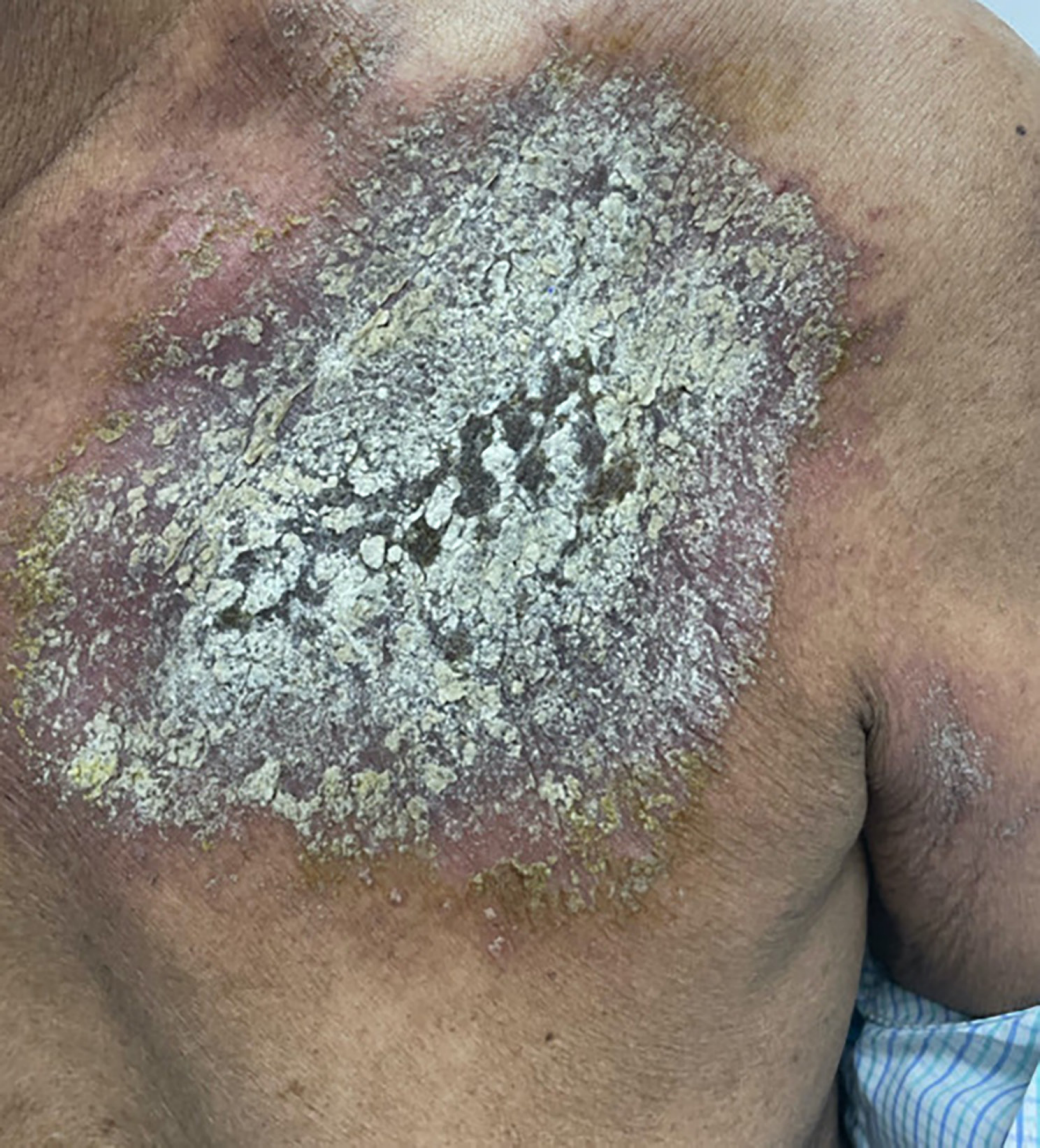
Erythematous, scaly, encrusted plaque engrained with topical remedies, with poorly defined borders, surrounding erythema over left upper anterior chest region.

**FIGURE 2:**
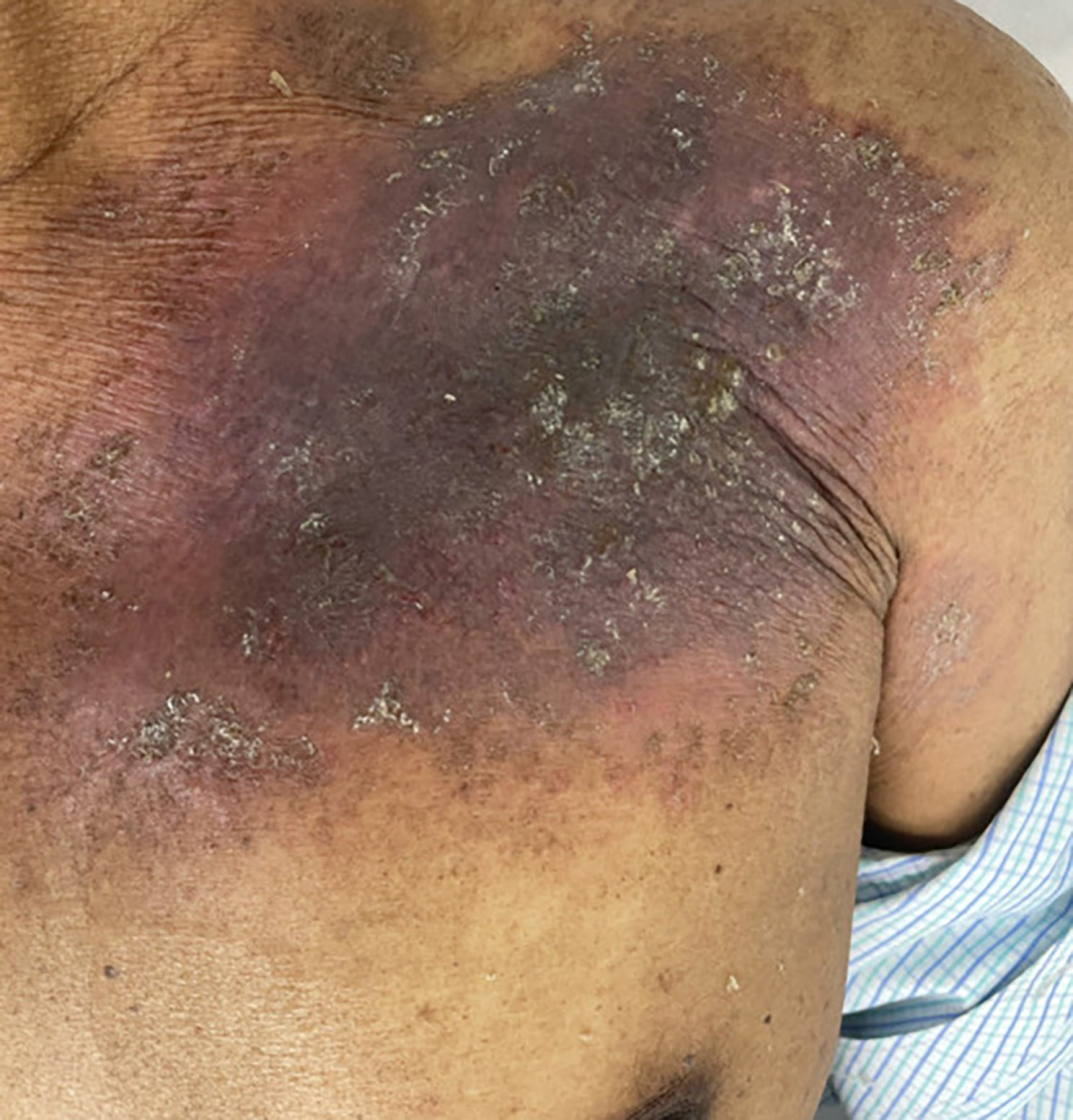
Erythematous plaque with sparse islands of crust, after scraping of overlying encrusted thick plaque in same patient.

A 33‐year‐old gentleman presented with a confluent dark papulosquamous lesion with poorly defined borders with surrounding erythema at the left infraclavicular region (Figure 1). On enquiry, he stated that it was present for 7 months after he met with a road traffic accident (RTA) and sustained some blunt injuries. After the RTA, he consulted some wandering quack and spiritual healer considering his accident as a bad omen. The spiritual healer cum quack prescribed topical herbal medications. The quack asked him not to apply water. After the first visit with the quack, he did not meet him again and did not apply water to the affected area for 7 months. In spite of the herbal medications, the pain in the left infraclavicular region persisted. Due to the pain, he consulted an orthopedician who on inspection found some cutaneous lesions and referred the case to us (dermatology outpatient department) for expert opinion. On scraping the skin, a thick dirt scale came out, which was present over a background of erythema, probably due to the underlying trauma, which did not heal properly (Figure 2). He was advised to use one topical antibiotic cream (fusidic acid 2%), a bland emollient (White soft Paraffin IP 13.2% W/V Light Liquid Paraffin IP 10.2% W/V in Lotion base). He was counseled properly and was adviced to regularly clean the body and the importance of cleanliness was explained. The case was then referred back to the orthopedician.

### 2.2 Case 2

**FIGURE 3:**
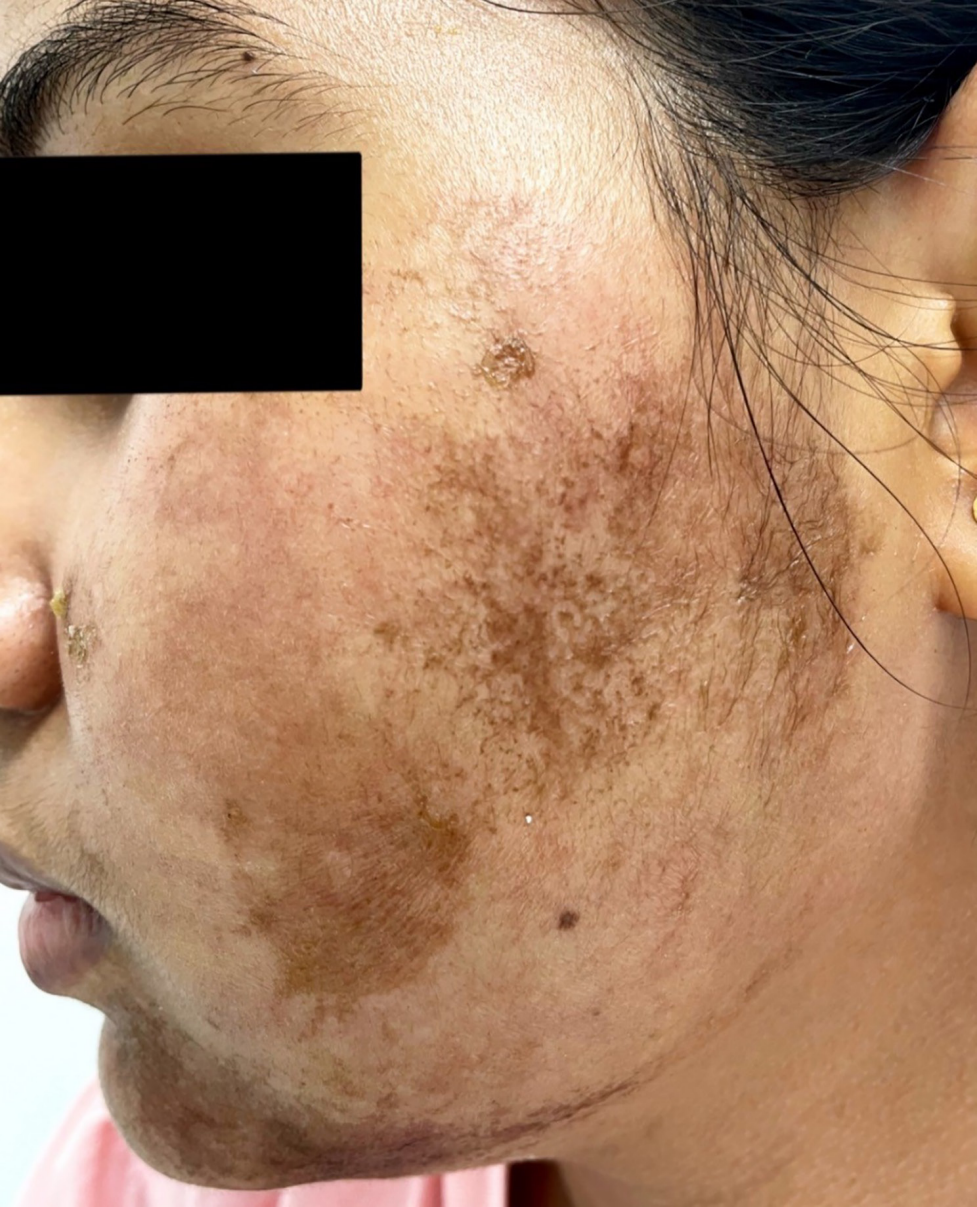
Erythematous plaque with adherent scales and brownish hue attributed to chemicals applied over left cheek, extending toward the chin.

**FIGURE 4:**
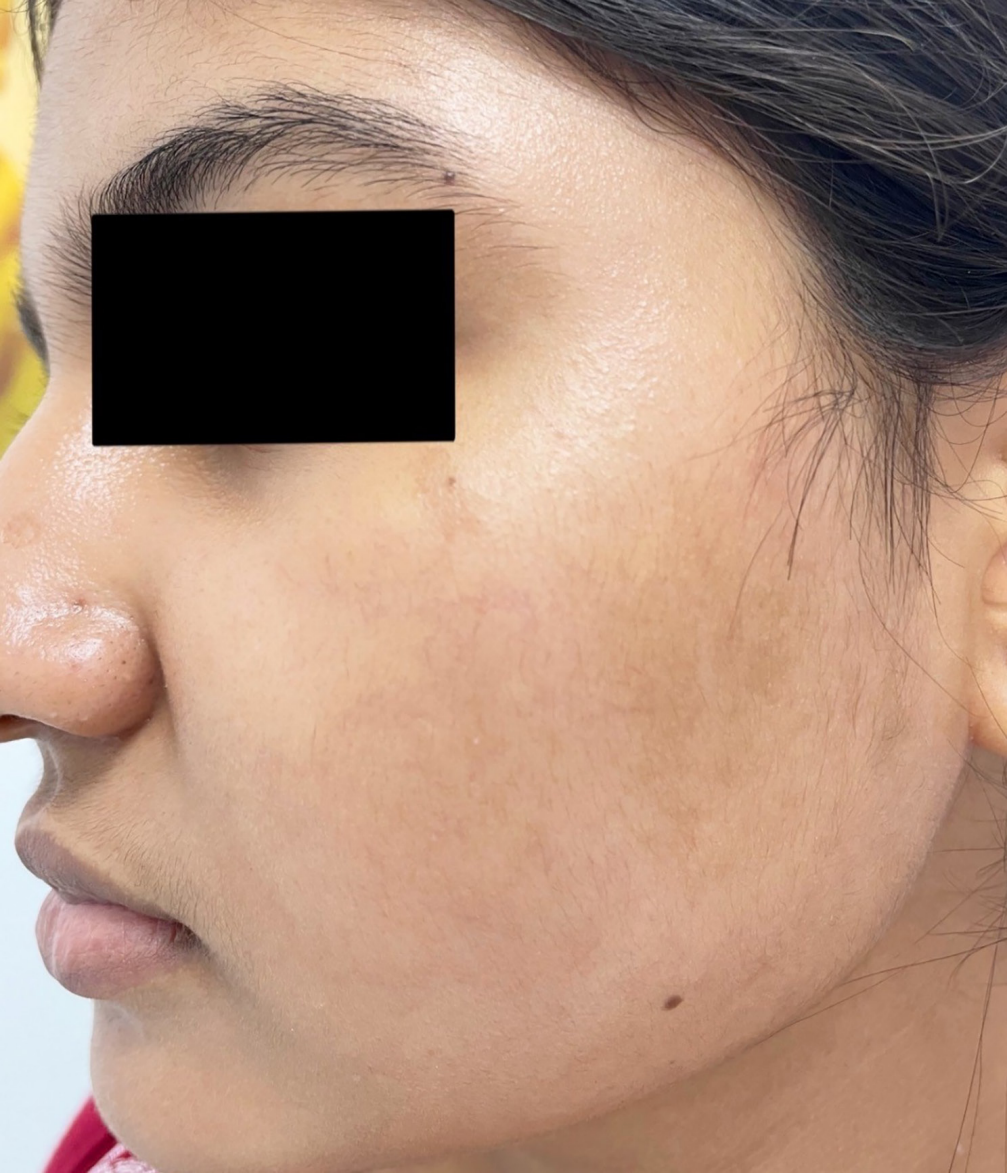
Normal appearing skin of same patient after saline wash exfoliation of scale and application of bland emollient.

A 23‐year‐old lady presented with dark adherent scales on her cheeks for 4 weeks (Figure 3). She said she visited some beauty parlor that advised her of unique facial rejuvenation techniques. She mixed the ingredients and used to apply the combination once a week. She was advised not to use any cleansing procedures. As an ardent follower of her (so‐called) beautician, she neither applied anything over her face nor used any cleansing products. At the end of 4 weeks, she developed pruritus and adherent scales. The guardians of the patient brought her for a dermatology consultation. After mild scraping, the scales started to come out. The face was soaked with a saline‐soaked gauge for 10 min, and then the scales became loose. The scales began to come out with gentle scraping. Then, a bland emollient was applied, and the skin became unremarkable (Figure 4). The patient was sent home with the advice to regular moisturizer (White soft Paraffin IP 13.2% W/V Light Liquid Paraffin IP 10.2% W/V in Lotion base) and to consult a certified dermatologist for every skin problem.

## 3 METHODS

The presented cases underscore the emerging concern of unscientific practices leading DA. Such scenario can aptly be termed as quack‐induced dermatitis (QID). This diagnostic category, analogous to dermatitis artefacta, is characterized by cutaneous lesions resulting from misguided treatments by non‐qualified practitioners or quacks. *The term “quack” is more appropriately applied to someone lacking a medical degree or proper qualifications who engages in the practice of medicine without valid credentials*. Quack‐induced dermatitis is a diagnosis of exclusion, requiring thorough evaluation through patients detailed history. It is imperative to differentiate QID from deliberate self‐harm, where patients are consciously involved in self‐inflicting habits. The management of QID involves proper counseling and the active treatment of lesions. Recognizing the impact of psycho‐social factors, such as underlying psychiatric conditions, anxiety, depression, phobia, and specially superstition, is essential, as these factors contribute to patients seeking unorthodox care and manupulation from quacks. This underscores the necessity for healthcare professionals to address not only the cutaneous manifestations but also the underlying psychological factors in the management of QID.

## 4 DISCUSSION

Dermatitis artefacta is a complex psychodermatological condition caused by underlying distress and its interplay with the sick role that patient portrays. Patients often deny their activity of self‐inflicting the cutaneous lesions. As patients are unaware that they are endangering their skin health, therapy should be primarily centered toward a multidisciplinary approach rather than defying the patient's concern. Dermatitis artefacta is a factitious skin disease, and several organic primary cutaneous mimickers can often mislead clinicians. The defying nature of this psychodermatological entity can be preceded by medical and, more often, underlying psychological conditions such as anxiety, phobia, or personality disorder. Often, patients lack any intent to gain rewards or gain of any sort patients. Dermatitis artefacta often encompasses the sickness role that a patient portrays to escape the social responsibility or, more often, to please one's psychological need. Causation of this factitious skin disorder is linked to prolonged personal or familial medical illness and psychosocial stress.^1^, ^2^

We would like to introduce the term “Quack Induced Dermatitis” for the family of dermatological disorders, which is produced due to unscientific treatment given by quacks in an otherwise normal skin.

The varied and bizarre clinical manifestation of this dermatological entity often confuses and misleads dermatologists to make any other primary cutaneous diagnosis as the final diagnosis. Even though patients are aware, they are not responsive to this harm caused to their skin. The varied clinical phenotype of this rare disease relies on various modes causing indeliberate harm. Morphology is often bizarre, not suggestive of any primary cutaneous dermatoses, and lesions in various stages can be present, from erosions and crusts to scars or pigmentation. Clinical morphology depends on the chemical “recipe” applied. Patients are often reluctant to share their history and can be anxious, depressed or have interpersonal conflicts. Quack‐induced dermatitis is always a diagnosis of exclusion such as DA and is often corroborated with history and clinical findings. It should be differentiated from deliberate self‐harms in patients, in which patients are conscious and aware of their self‐inflicting habits. Treatment of QID involves proper counseling and management of active lesion. Using emollients, topical and systemic antibiotics should be done concurrently. Underlying psychiatric anxiety, depression, phobia, or interpersonal conflict if present should be appropriately assessed and treated as social factors plays an important role in dragging patients to quacks. All these approaches help in the holistic treatment of patients with an ultimate efficient doctor–patient relationship, which adds to the most significant therapeutic benefits to patients. Regular follow‐up entailed by an excellent doctor–patient relationship is an essential aspect of treating this entity.^3^, ^4^

## 5 CONCLUSION

Both the patients have been symptom‐free post treatment and on regular follow‐up till date.

Dermatitis artefacta reveals varied clinical presentations that may lead to delayed diagnosis and cutaneous complications. Healthcare professionals, particularly dermatologists, should prioritize taking thorough patient histories to rule out this entity. These two case report illustrates two instances of DA, which was induced by unscientific advise and patient handling by quacks. It emphasizing the importance of repercussions of unscientific and harmful medical guidance from non‐qualified practitioners. Proper counseling and fostering a good doctor–patient relationship are crucial in preventing DA resulting from unscientific approaches.

## AUTHOR CONTRIBUTIONS

**Prajwal Pudasaini:** Conceptualization; formal analysis; investigation; methodology; writing – original draft. **Sadiksha Adhikari:** Formal analysis; methodology; writing – original draft. **Kinnor Das:** Investigation; project administration; writing – original draft. **Suraljit Gorai:** Investigation; methodology; writing – original draft; writing – review and editing. **Ruri D. Pamela:** Conceptualization; writing – original draft; writing – review and editing.

## FUNDING INFORMATION

None.

## CONFLICT OF INTEREST STATEMENT

None.

## CONSENT

Written informed consent was obtained from the patient to publish this report in accordance with the journal's patient consent policy.

## Data Availability

The data that support the findings of this study are openly available in Clinical Case Reports.
